# Autophagy-Mediated Adaptation: Revealing the Role of Autophagy in Plant Responses to Abiotic Stress

**DOI:** 10.3390/genes16121461

**Published:** 2025-12-07

**Authors:** Zixuan Yu, Abdul Waheed, Daoyuan Zhang, Asigul Ismayil, Yakupjan Haxim

**Affiliations:** 1 College of Life Sciences, Shihezi University, Shihezi 832000, China; yuzixuan00k@163.com; 2 National Key Laboratory of Ecological Security and Sustainable Development in Arid Areas, Chinese Academy of Sciences, Urumqi 800311, China; drwaheed@ms.xjb.ac.cn (A.W.);; 3 State Key Laboratory of Desert and Oasis Ecology, Xinjiang Institute of Geography and Ecology, Chinese Academy of Sciences, Urumqi 830011, China; 4 Xinjiang Key Laboratory of Conservation and Utilization of Plant Gene Resources, Xinjiang Institute of Ecology and Geography, Chinese Academy of Sciences, Urumqi 830011, China; 5 Turpan Eremophytes Botanical Garden, Chinese Academy of Sciences, Turpan 838008, China; 6 China-Tajikistan Belt and Road Joint Laboratory on Biodiversity Conservation and Sustainable Use, Xinjiang Institute of Ecology and Geography, Chinese Academy of Sciences, Urumqi 830011, China

**Keywords:** autophagy, abiotic stress, autophagy-related genes, molecular mechanisms, crop resilience

## Abstract

Autophagy, an evolutionarily conserved intracellular recycling pathway, is essential for maintaining cellular homeostasis and enhancing plant resilience to a variety of abiotic stresses, including drought, salinity, extreme temperatures, and heavy metal toxicity. Be-yond its canonical role in nutrient recycling, autophagy is now recognized as a central regulator of stress signaling, hormonal crosstalk, and metabolic reprogramming. Here we synthesize the functions of autophagy under diverse abiotic stresses, highlighting its role in organellar quality control, metabolic adaptation, and stress-specific responses. We further discuss innovative strategies for enhancing crop resilience, including genome editing, integrative multi-omics analyses, and synthetic biology applications. Elucidating the autophagy regulatory network provides the foundation for designing next-generation crops that maintain high yield and resilience under climate-driven stress.

## 1. Introduction

As sessile organisms, plants are continuously exposed to abiotic stresses such as drought, salinity, extreme temperatures, nutrient deficiency, and heavy metal toxicity [1,2]. These stresses severely constrain plant growth, development, and productivity. To cope with adverse conditions, plants have evolved highly integrated environmental sensing and response networks. Among these, autophagy—an evolutionarily conserved intracellular degradation pathway—plays an important role. Autophagy mediates the delivery of damaged organelles, protein aggregates, and other cytoplasmic components to the vacuole/lysosome for degradation via the formation of double-membrane autophagosomes, thereby recycling cellular materials and redistributing energy [3]. This process is not only crucial during physiological stages such as seed germination, leaf senescence, and nutrient remobilization, but is also particularly important in responses to environmental stress [4,5,6].

With advances in molecular biology and multi-omics approaches, the core mechanisms and regulatory networks of plant autophagy have become increasingly well characterized. Evidence indicates that autophagy modulates cellular homeostasis, metabolic pathways, and stress signaling, playing a key role in plant adaptation to abiotic stress. This review aims to systematically summarize recent progress, elucidate the functions and molecular bases of autophagy under different stresses, and discuss its potential applications and future challenges in crop stress resilience breeding.

## 2. Core Machinery and Physiological Roles of Plant Autophagy

**Figure 1:**
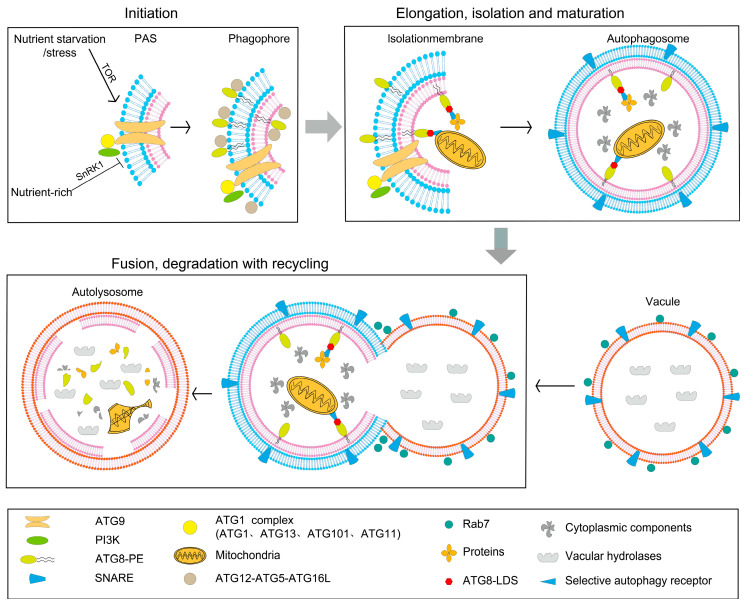
Overview of autophagy process in plants. Initiation, where the ATG1 and PI3K complexes are activated at the PAS to initiate autophagy; Elongation, Isolation, and Maturation, during which the isolation membrane extends with the help of the ATG12-ATG5-ATG16L complex and the ATG8-PE conjugation system; Fusion, Degradation with Recycling, where mature autophagosomes fuse with the vacuole. This fusion is mediated by SNARE proteins and Rab GTPases, which lead to the degradation and recycling of cellular components within the vacuole.

Plant autophagy is a conserved catabolic pathway driven by over 40 ATG (autophagy-related) proteins [7]. It encapsulates damaged organelles, protein aggregates, and other cytoplasmic components within double-membrane autophagosomes and delivers them to the vacuole for degradation and recycling [3,7]. This process is essential not only for nutrient recycling but also plays broad roles in plant growth, development, and adaptation to environmental stresses [8,9] (Figure 1).

### 2.1. The Core Machinery Stages of Autophagosome Formation

The autophagic process can be divided into four sequential stages: initiation, nucleation and elongation, maturation and fusion, and degradation with recycling. These steps are orchestrated by the evolutionarily conserved ATG proteins, whose concerted action drives autophagosome formation, cargo recognition and vacuolar degradation [7].

Initiation—Plants continuously sense nutrient and energy availability to modulate autophagic flux. The target-of-rapamycin (TOR) kinase and sucrose-non-fermenting-1-related kinase 1 (SnRK1) act antagonistically: TOR actively represses autophagy under energy-replete conditions, whereas SnRK1 triggers the program upon carbon or energy deficit and multiple abiotic stresses [10]. Downstream of stress cues, the ATG1–ATG13 protein kinase complex (comprising ATG1, ATG13, ATG101 and ATG11) is recruited to the phagophore-assembly site (PAS). There it cooperates with the transmembrane protein ATG9, which shuttles between the endomembrane compartments, to promote phagophore nucleation and expansion, thereby committing the cell to autophagosome biogenesis [11]. Loss-of-function alleles of ATG13 in Arabidopsis produce a strong autophagy-defective phenotype and hypersensitivity to starvation, underscoring the centrality of this complex in autophagy induction [12]. The class-III phosphatidylinositol 3-kinase (PI3K) complex, which generates phosphatidylinositol 3-phosphate (PI3P), functions as an additional master regulator that recruits PI3P-binding effectors and early autophagic membranes to the PAS [13].

Nucleation and elongation: During nucleation, PI3K complex—composed of ATG6 (the Arabidopsis VPS30 orthologue), ATG14 and VPS34—synthesizes PI3P, an essential lipid that initiates phagophore formation in plants [7,14]. PI3P recruits PI3P-binding effectors such as ATG18 to support membrane expansion and provides a spatial cue for selective autophagy receptors, ensuring correct cargo loading and autophosome assembly [7]. Elongation proceeds via two interconnected ubiquitin-like conjugation systems: the ATG12–ATG5–ATG16L complex acts as an E3-like ligase to drive phagophore curvature, while ATG8 is covalently conjugated to phosphatidylethanolamine (PE). The resulting ATG8–PE adduct acts as a membrane scaffold and docking platform for selective receptors, thereby powering cargo sequestration and autophosome closure [15,16]. The PI3P effector ATG18 further stabilizes the expanding membrane and facilitates its sculpting into a sealed double-membrane vesicle [17].

Maturation and fusion: Autophagosomes are delivered to the vacuole through SNARE-mediated tethering and Rab-GTPase-dependent membrane fusion. Syntaxin 17 (STX17) and Rab7 coordinate the docking and merger of the outer autophagosomal membrane with the tonoplast, releasing the autophagic body into the lytic vacuolar lumen [18,19].

Degradation and recycling: Vacuolar hydrolases rapidly dismantle the autophagic body, liberating amino acids, fatty acids, sugars and cofactors that are re-imported into the cytosol to sustain primary metabolism and stress adaptation.

### 2.2. Distinctive Features of Autophagy in Plants

The overall framework of autophagy is highly conserved across eukaryotes, encompassing phagophore initiation, autophagosome expansion and closure, and subsequent fusion with the lysosome or vacuole for degradation of cytoplasmic components [3,20]. However, plants exhibit distinct molecular features and regulatory mechanisms.

One unique aspect is the diversity of ATG8 isoforms in plants. In contrast, although mammals also possess a small ATG8 protein family comprising several subtypes such as LC3 and GABARAP, the number and diversity of their isoforms are substantially lower than those in plants. This diversity may provide plants with a broader capacity for cargo selectivity and regulatory complexity [21]. Moreover, structural differences in the ATG1 kinase complex between plants and animals suggest that its signaling roles are not entirely conserved.

At the upstream regulatory level, mammalian autophagy is primarily controlled by antagonistic interactions between the mammalian target of rapamycin (mTOR) and AMP-activated protein kinase (AMPK): mTOR inhibits autophagy, while AMPK activates it [22]. SnRK1 acts upstream of TOR kinase to activate autophagy in *Arabidopsis*, establishing TOR as a core negative regulator. The discovery of TOR-independent activation pathways, notably under oxidative and endoplasmic-reticulum (ER) stress, reveals a greater complexity in the plant autophagy regulatory network, the full details of which are not yet resolved. [23]. Accumulating evidence indicates that plants enhance autophagosome formation under stress conditions by repressing TOR activity, thereby strengthening their adaptive capacity [24].

Overall, although the core machinery of autophagy is conserved between plants and animals, the unique molecular components and regulatory modes in plants reflect evolutionary adaptations to fluctuating environments. Comparative analyses of autophagy across systems not only shed light on its functional diversification during evolution but also provide a theoretical basis for enhancing crop stress tolerance.

### 2.3. Physiological Functions of Autophagy: From Growth and Development to Stress Adaptation

Autophagy, a conserved intracellular degradation process, contributes to the selective removal of damaged organelles, misfolded proteins, and other harmful components [5]. By maintaining cellular homeostasis, autophagy plays pivotal roles in plant growth, development, and stress adaptation, thereby ensuring survival and fitness under adverse conditions [6].

#### 2.3.1. Seed Germination and Seedling Establishment

Basal levels of autophagy are essential under normal growth conditions to remove damaged organelles and proteins, thereby supporting cellular homeostasis and energy metabolism [9]. During seed germination and seedling establishment, autophagy contributes to the mobilization of storage proteins and lipids, supplying energy and carbon sources to sustain early growth [25]. Autophagy-deficient mutants typically exhibit delayed germination and impaired seedling development [26]. In addition, autophagy participates in nutrient remobilization and hormone-mediated developmental regulation, including pathways governed by auxin and brassinosteroids, thereby influencing organ development and growth dynamics [27].

#### 2.3.2. Nutrient Remobilization and Delay of Senescence

Autophagy mitigates cellular senescence by removing dysfunctional chloroplasts and mitochondria, thereby limiting the excessive accumulation of reactive oxygen species (ROS) [28]. In parallel, autophagy facilitates nutrient remobilization from senescing organs to actively growing tissues, a process that supports plant growth under nutrient-limiting or stressful conditions [29,30]. This dual role highlights autophagy as a critical regulator of both cellular quality control and whole-plant resource allocation [31].

#### 2.3.3. Stress Memory and Transgenerational Epigenetic Regulation

Emerging evidence suggests that autophagy functions not only as an immediate stress-response mechanism but may also contribute to stress memory and transgenerational epigenetic regulation [32]. Such findings provide novel insights into the long-term adaptive capacity of plants and open new avenues for understanding the evolutionary and breeding potential of autophagy-mediated processes.

## 3. Autophagy as a Central Integrator of Abiotic Stresses Tolerance

**Figure 2:**
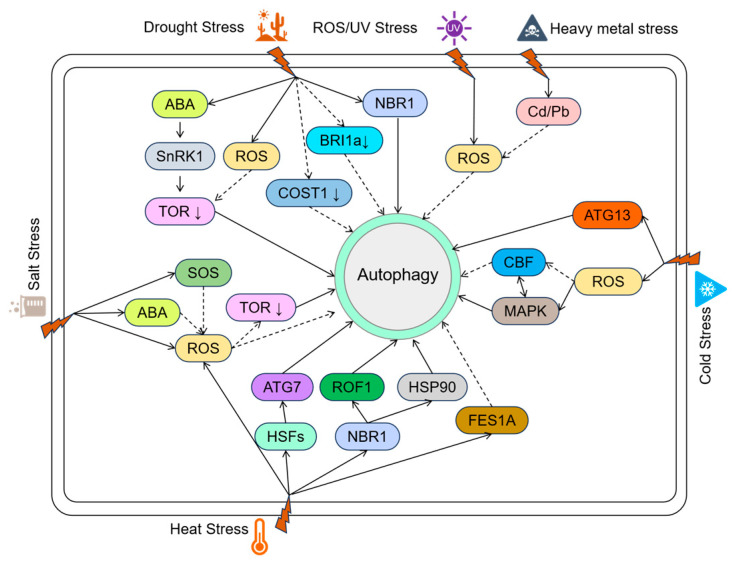
Autophagic Pathways Induced by Different Abiotic Stresses in Plants.

Abiotic stresses such as drought, salinity, extreme temperatures, high light, and heavy metal toxicity severely constrain plant growth and productivity. To cope with these challenges, plants have evolved diverse adaptive mechanisms, among which autophagy plays a central role. As a highly conserved degradation and recycling process, autophagy contributes to plant stress tolerance by removing damaged organelles and proteins, maintaining cellular homeostasis, and reallocating resources (Figure 2).

Recent reviews highlight that autophagy acts as a central integrator of multiple abiotic stress signals, coordinating organelle quality control, redox balance, and metabolic adaptation. Li et al. (2023) summarized regulatory networks of autophagy under diverse stresses and suggested its potential for enhancing crop resilience [33]. Recent reviews suggest that integrating autophagy pathways into breeding and biotechnology can significantly enhance crop stress tolerance [34]. Below, we summarize current insights into the roles and regulatory mechanisms of autophagy under major abiotic stresses.

### 3.1. Drought Stress

Drought is a primary environmental constraint limiting plant growth and yield. Studies in Arabidopsis have demonstrated that water deficit rapidly activates autophagy; conversely, *atg5*, *atg7* and other autophagy-defective mutants exhibit severely reduced survival under limited irrigation, whereas over-expression of *ATG* genes such as apple *MdATG18a* significantly improves drought tolerance [35,36].

Autophagy induction is orchestrated by the convergent action of abscisic acid (ABA), ROS and the TOR kinase. ABA relieves TOR repression via the SnRK1-TOR cascade and directly phosphorylates ATG1/ATG13 through SnRK2, thereby promoting autophagosome nucleation [37]. Parallel ROS bursts oxidize ATG4 cysteine residues, accelerate ATG8–PE conjugation and modulate selective receptors such as NBR1 to eliminate damaged mitochondria and peroxisomes, thus attenuating oxidative injury and preserving cellular homeostasis. Drought-evoked ER stress activates the IRE1–bZIP60 pathway, which up-regulates ATG transcription to alleviate ER overload and intersects with the broader autophagy regulatory network. WRKY53 and TGA transcription factors bind ATG promoters in response to oxidative stress, sustaining ATG expression and autophagic flux under dehydration [38].

**Figure 3:**
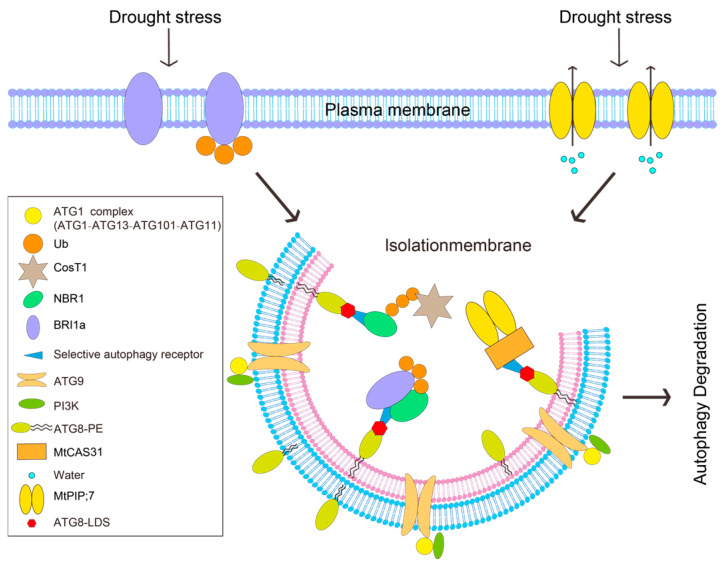
Autophagy in Response to Drought. Under drought stress, the autophagy receptor NBR1 enhances drought tolerance by upregulating the expression of autophagy-related genes and facilitating the selective autophagic degradation of the negative regulator BRI1a. Additionally, autophagy can be activated by the degradation of the autophagy negative regulator COST1, and it can also improve survival rates by degrading the negative regulator *MtPIP2;7*, thereby reducing water loss.

Once activated, autophagy removes impaired mitochondria and peroxisomes, blunts ROS escalation and degrades aquaporins such as *MtPIP2;7* to limit water loss, thereby maintaining both cellular water potential and redox balance [39]. Recent work has identified mitochondrial alternative oxidase 1a (AOX1a) as a critical node in this circuitry: drought-induced ethylene signals, acting through EIN3/EIL1, up-regulate AOX1a, restrain mitochondrial ROS over-accumulation and activate PINK1–Parkin-mediated mitophagy that selectively eliminates dysfunctional mitochondria and recycles amino acids and fatty acids for osmotic adjustment and energy replenishment, markedly increasing drought survival in tomato and other crops. AOX1a over-expression or ethylene treatment elevates LC3-II/ATG8-PE levels and accelerates p62 turnover, whereas autophagy inhibition abolishes the ethylene-conferred drought tolerance, thus establishing a “drought–ethylene–AOX–autophagy” tripartite axis that operates alongside the canonical ABA–SnRK1–TOR pathway to integrate hormonal cues, energy status and organelle quality under water deficit [40]. *COST1*, a plant-specific negative regulator of autophagy, suppresses drought tolerance under favorable conditions; drought-triggered *COST1* degradation relieves this brake, enabling autophagy activation at the cost of growth but enhancing survival [41] (Figure 3).

Autophagy contributes to drought tolerance in a species-specific manner. In Arabidopsis, autophagy activation is indispensable for coping with water deficit: atg5 and atg7 mutants suffer more severe cellular damage and a pronounced drop in survival under drought [38]. Apple plants over-expressing *MdATG18a* exhibit improved water-use efficiency and drought endurance, illustrating the agronomic potential of manipulating autophagy in perennial fruit crops [36]. Maize autophagy receptor *ZmNBR1* enhances drought tolerance by up-regulating ATG genes and selectively degrading the negative regulator *ZmBRI1a*; *ZmNBR1* thereby clears damaged organelles and sustains autophagic flux, boosting whole-plant survival under limited water availability [42]. Wheat benefits from drought-induced autophagy through mitophagy that removes ROS-compromised mitochondria and safeguards energy supply, resulting in stronger drought resilience [43]. In *Caragana korshinskii*, drought markedly up-regulates *ATG8* paralogues whose expression levels correlate positively with sucrose and trehalose accumulation, indicating that autophagy modulates carbohydrate metabolism to maintain energy balance and cellular stability [44]. Potato *StATG8a* is sharply induced by drought and cooperates with ABA/JA signaling to scavenge Na^+^, ROS and protein aggregates, preserve chloroplast and mitochondrial integrity, and optimize stomatal behavior, collectively elevating drought tolerance [45]. Over-expression of TdAtg8 from wild emmer wheat improves drought performance via simultaneous mitophagy and lipophagy that eliminate injured organelles and lipid bodies, thereby sustaining cellular homeostasis [46]. *SiATG8a* from *Setaria italica* (Foxtail millet) is strongly induced under combined low-nitrogen and drought stress; its ectopic expression in Arabidopsis enhances drought endurance by promoting both lipophagy and ribophagy to remobilize storage lipids and ribosomal proteins, maintaining resource equilibrium under water deficit [47].

### 3.2. Cold Stress

Low temperatures, particularly freezing episodes, constitute a major threat to plant growth and survival. To counteract this stress, plants deploy a suite of physiological mechanisms that preserve intracellular homeostasis, among which autophagy is emerging as a critical response pathway. Chilling stress rapidly triggers autophagic flux that removes damaged organelles and misfolded proteins, thereby sustaining cellular integrity. The initial sensing step involves the transcriptional activation of core autophagy genes such as *OsATG8* and *OsATG5*; gene-co-expression network analyses in rice have identified chilling-enriched modules that are highly populated by autophagy-related pathways, underscoring the pivotal role of autophagy in low-temperature acclimation [48]. Specifically, execution genes including *OsATG8a* and *OsATG12* are markedly up-regulated upon cold exposure, providing the molecular framework that enables plants to adapt to sub-optimal thermal environments.

Moreover, low temperatures trigger autophagic flux through multiple convergent pathways. Chilling impairs mitochondrial integrity, provoking ROS accumulation and a drop in membrane potential that selectively activates ATG8-dependent mitophagy to eliminate dysfunctional mitochondria and preserve cellular energetics. In rice, cold-induced mitochondrial damage is sensed by BNIP3-like receptors that tag impaired organelles for autophagic clearance, thereby attenuating chilling injury. Concomitantly, the unfolded-protein response (UPR) is engaged: cold stress up-regulates *OsFAM134B* and related ER-phagy receptors that deliver misfolded proteins to the vacuole, relieving ER overload [49]. In alfalfa, *MsATG13* is transcriptionally induced by cold and boosts autophagic activity, which in turn reinforces the antioxidant machinery to scavenge ROS and mitigate oxidative damage [50]. Collectively, these findings reveal that autophagy not only executes direct damage repair but also intersects with ROS homeostasis and energy metabolism, enabling plants to maintain growth and survive under sub-optimal thermal conditions.

### 3.3. Salt Stress

Soil salinity is a major abiotic constraint limiting plant growth and global crop productivity. Salt stress elicits ionic imbalance, osmotic perturbation and excessive ROS, culminating in oxidative damage and ER stress. To counteract these insults, plants activate multiple adaptive pathways among which autophagy occupies a central role in preserving cellular homeostasis and stress tolerance [51,52,53,54].

Salt-induced autophagy is orchestrated by a complex signaling network that integrates ABA, ROS and the TOR kinase. ABA-mediated inhibition of TOR activity promotes autophagic flux, thereby enhancing tolerance to osmotic and oxidative challenges [55]. Arabidopsis *atg5* and *atg7* mutants accumulate excess ROS and exhibit aggravated cell death under salinity, underscoring the protective function of autophagy in redox balance [56]. Similarly, silencing of *TaATG2* and *TaATG7* in wheat suppresses autophagosome formation and intensifies programmed cell death [57], whereas the metacaspase TaMCA-Id negatively regulates salt-induced cell death via functional crosstalk with the autophagy pathway [58], revealing an intricate antagonism between autophagy and cell-death programs during salt stress.

Recent studies have uncovered the molecular framework underlying salt-stress-induced selective autophagy, with particular emphasis on endoplasmic-reticulum autophagy (ER-phagy). Salinity provokes protein misfolding within the ER and activates the UPR; to alleviate ER stress, plants selectively deliver damaged ER domains and aberrant proteins to the vacuole via ER-phagy. This process is controlled by the ubiquitin-fold modifier 1 (Ufm1) pathway: under saline conditions the Ufm1-activating and E3-ligating enzyme Ufl1 catalyzes ufmylation of specific ER-resident or misfolded proteins, thereby tagging them for autophagic recognition and clearance. Ufl1-mediated ER-phagy enables plants to eliminate misfolded cargo, restore ER homeostasis and attenuate chronic UPR signaling. The discovery establishes a direct molecular link between the Ufm1 system and autophagy-dependent proteostasis, offering new insight into how plants integrate protein-quality control with stress adaptation [59].

In addition to the endoplasmic reticulum, other organelles participate actively in the autophagic response to salt stress. Peroxisomes exhibit dynamic numerical and morphological changes under salinity and in autophagy-defective backgrounds. Real-time imaging with the N-BODIPY fluorescent probe revealed that the number of peroxisomes increased markedly in wild-type Arabidopsis leaves after NaCl treatment, whereas salt-hypersensitive mutants such as fry1 and sos1 failed to display this proliferation. Conversely, the autophagy-defective mutant atg5-1 showed aberrant peroxisome accumulation, indicating that autophagy—specifically peroxisome autophagy (pexophagy)—is essential for peroxisomal homeostasis during salt stress [51]. Thus, salinity simultaneously stimulates peroxisome biogenesis and pexophagic degradation, thereby coordinating ROS metabolism and cellular detoxification to maintain redox equilibrium.

Multi-species studies have further consolidated the universal role of autophagy in salt tolerance. In cotton, salinity strongly up-regulates *GhATG8f*; over-expression lines show higher survival and photosynthetic activity, whereas RNAi-mediated silencing reduces salt tolerance, indicating that *GhATG8f*-dependent autophagy clears damaged organelles and sustains ionic homeostasis under NaCl stress [60]. Over-expression of BnaA8.ATG8F in rapeseed enhances both autophagosome formation and vacuolar degradation, improving plant performance under combined salt and nitrogen limitation [61]. Apple plants over-expressing *MdATG10* exhibit elevated autophagic flux that maintains cellular homeostasis and increases salt endurance [62], while in poplar the selective autophagy receptor NBR1 removes salt-damaged organelles and simultaneously boosts antioxidant enzymes (SOD, CAT, GPX), thereby reinforcing salt-stress tolerance [63].

Interplay between regulatory factors and signaling pathways further shapes the autophagic response. Under salinity, the apple MdLRR-RLK1–MdATG3 module and the transcription factor MdHB7-like promote autophagosome formation and Na^+^ efflux to augment salt resistance [64,65]. In mulberry, over-expression of an RGS protein negatively regulates salt tolerance by increasing ROS accumulation; concomitant up-regulation of *MaATG7* and *MaATG8* is thought to mitigate this oxidative burden via enhanced autophagy [66]. Spermidine application in cucumber activates H_2_O_2_-mediated autophagy that alleviates salt-induced oxidative damage [67].

Collectively, these findings demonstrate that salt-triggered autophagy not only eliminates injured organelles (ER and peroxisomes) but also integrates with antioxidant systems, ionic homeostasis and hormonal signaling. Although species-specific nuances exist, autophagy operates as a conserved, multi-layered hub that governs cellular homeostasis during salt stress.

### 3.4. Heat Stress

Heat stress triggers protein-misfolding, oxidative burst and organelle damage in plant cells, forcing them to mount a suite of protective responses. Central among these is autophagy, which maintains cellular homeostasis by removing injured organelles and aggregated proteins [68]. A large body of work has linked autophagic flux to thermotolerance. Under heat stress, an atypical conjugation of ATG8—operating independently of the canonical E1-E2 enzymes ATG7 and ATG3—boosts autophagosome biogenesis, presumably by modulating mTOR signaling and/or alternative ligases, thereby enhancing heat survival [69].

In Arabidopsis, acute heat drives key autophagy proteins into stress granules; this sequestration transiently restrains autophagy during stress but primes its rapid re-activation during recovery to clear ubiquitinated protein aggregates, ultimately strengthening thermotolerance [70]. Autophagy also operates beyond the cell scale: in the C3-C4 intermediate Moricandia suffruticosa, combined heat and drought increase leaf vein density, a response that coincides with autophagy-associated vesicle trafficking and altered expression of autophagy-related genes, implying that autophagy helps re-engineer leaf anatomy to sustain photosynthesis under thermal stress [71].

High temperatures (40–43 °C) fine-tune autophagy through the Ca^2+^-calpain axis. Calpain-mediated cleavage of ATG5 and associated regulators (e.g., Gα subunits) gates autophagy initiation and intersects with cell-death pathways to balance survival during heat stress. Heat-shock transcription factors (HSFs) add transcriptional control: tomato HsfA1a directly binds promoters of ATG10 and ATG18f to up-regulate their expression, reinforcing autophagic flux and maintaining both proteostasis and ROS homeostasis. Arabidopsis *HSFA1s* (*a*, *b*, *d*, *e*) perform an equivalent function by activating the broader heat-shock response (HSR) gene network [72].

Recovery from heat stress is equally critical. Autophagy degrades excess stress molecules such as *HSPs* and *ROF1*, resetting cellular memory and priming plants for subsequent heat episodes [73]. The plastid metalloprotease *FtsH6* normally removes *HSP21* during recovery; in ftsh6 mutants, autophagy compensates by clearing *HSP21*, illustrating functional crosstalk between protease systems [74]. Selective autophagy also targets the TOC translocon at the chloroplast outer envelope, attenuating protein import and adjusting photosynthetic capacity. The autophagy receptor NBR1 recognizes ubiquitinated chloroplast proteins and facilitates their disposal, thereby sustaining thermotolerance [69].

NBR1-mediated selective autophagy further curbs hyper-responses during recovery by degrading *HSP90.1* and *ROF1*, which dampens HSFA2-driven transcription of heat-shock genes. nbr1 mutants retain stronger heat-stress memory under recurring heat, underscoring NBR1’s role in mitigating over-reaction to repeated stress [32]. NBR1 also participates in Golgi re-assembly after heat-induced disorganization, accelerating the return to homeostasis [75].

Transgenic evidence across species corroborates these findings. Over-expression of the sunflower protein HaFT-1 in Arabidopsis elevates autophagic flux, clears heat-damaged proteins/organelles and improves survival [76]. Likewise, tomato plants over-expressing SlATG8f display higher autophagosome abundance, better preserved pollen morphology and elevated pollen viability after heat treatment; co-over-expression of *SlMDH3* with its interactor *SlATI1* further reduces heat-induced cellular damage and boosts antioxidant enzyme activity, suggesting that the MDH3-ATI1 module promotes cytoprotection via autophagy [77,78].

In apple, *MdATG18a* over-expression accelerates autophagic flux (increased LC3-II, abundant autophagic bodies) and speeds clearance of heat-injured chloroplasts, limiting ROS accumulation and thylakoid disintegration, thereby safeguarding photosynthetic integrity and basal thermotolerance [79]. Potato *StATG18a* acts similarly: over-expression enhances antioxidant capacity and photosynthetic performance, whereas *StATG18a* knock-down lines suffer aggravated oxidative damage under heat, confirming the gene’s protective role [80].

Collectively, these studies provide a mechanistic framework showing that autophagy is indispensable for heat-stress adaptation across diverse crops, and they offer gene-editing and transgenic strategies for engineering improved thermotolerance in an era of escalating global temperatures.

### 3.5. Heavy Metal Stress

Heavy metal toxicity—particularly from cadmium (Cd) and lead—poses a major threat to plant performance and food safety. Within minutes of Cd or other metal exposure, autophagy is rapidly engaged; integrated transcriptome and proteome surveys consistently rank the *ATG3/5/7/8/12/18* module and Beclin-1 orthologues among the most highly induced pathways. Different metals exhibit distinct selectivity: Cd preferentially drives the ATG8–PE conjugation system, arsenic (As) primarily activates the ER-phagy receptor *FAM134B*, whereas nickel (Ni) chiefly recruits PINK1/Parkin-mediated mitophagy [81]. At sub-lethal doses, metal-generated ROS activate the AMPK–ULK1 axis and suppress mTOR, promoting autophagosome biogenesis that, in turn, removes ROS-generating organelles (damaged mitochondria and peroxisomes) and re-establishes ion homeostasis. By contrast, prolonged or high-dose exposure impairs lysosomal acidification (down-regulation of LAMP-2), leading to autophagosome accumulation, a concomitant rise in both p62 and LC3-II, and a terminal switch to programmed cell death [82]. Cadmium further amplifies oxidative injury via NCOA4-mediated ferritinophagy, which liberates Fe^2+^ to fuel lipid peroxidation that synergizes with mitophagy-driven mitochondrial collapse [83].

In Arabidopsis, Cd challenge triggers HIPP33-directed selective autophagy that sequesters Cd in the vacuole, markedly lowering cytosolic toxicity [84]. Apple plants over-expressing MdATG10 display accelerated clearance of injured organelles, enhanced redox homeostasis and significantly improved Cd tolerance [85]; conversely, atg5 and atg7 mutants accumulate higher levels of toxic ions and exhibit hypersensitivity [86].

Collectively, these findings demonstrate that autophagy assembles a multi-layered protective network under heavy metal stress—operating through ion sequestration, removal of damaged organelles and metabolic reprogramming—whose intensity and duration ultimately dictate whether the plant adapts or succumbs to metal toxicity [6,87].

### 3.6. Nutrient Starvation

Nutrient limitation is a widespread abiotic stress factor constraining crop productivity. Autophagy plays a pivotal role in nutrient recycling during starvation, allowing cells to reutilize macromolecules and maintain metabolic homeostasis [9]. Under nitrogen, phosphorus, carbon, and other essential nutrients, autophagy mobilizes intracellular resources to sustain metabolism and growth, thereby improving nutrient-use efficiency [88,89,90].

Specifically, during nitrogen starvation, autophagy is indispensable for nitrogen remobilization from senescing leaves to developing tissues. In maize, the atg12 mutant exhibits compromised autophagic flux, pronounced growth retardation under nitrogen-deficient conditions, premature leaf senescence, and a markedly reduced seed nitrogen harvest index, underscoring the essential role of autophagy in nitrogen economy [91]. Consistent phenotypes are observed in the rice mutant Osatg7-1, which displays reduced biomass accumulation, impaired nitrogen retranslocation, and early vegetative senescence [30]. Manipulation of ATG genes further demonstrates the potential of autophagy in improving nutrient utilization: overexpression of *OsATG8b* significantly enhances nitrogen remobilization efficiency and grain quality under low-nitrogen supply, whereas RNAi-mediated suppression of this gene results in diminished nitrogen recycling capacity [92].

Autophagy is also robustly activated under carbon starvation. In Arabidopsis, carbon depletion induces extensive autophagosome formation and vacuolar degradation of chloroplast stroma proteins, thereby supplying amino acids and carbon skeletons required to sustain respiration and tricarboxylic acid (TCA) cycle fluxes. Autophagy-defective mutants such as atg5 show profound metabolic imbalance, accumulation of damaged organelles, and rapid mortality during extended darkness, demonstrating that autophagy is essential for maintaining energy homeostasis under carbon-limited conditions [9].

Under phosphate (Pi) starvation, autophagy contributes to Pi conservation through selective ER-phagy. Iron-mediated ER stress rapidly activates ER-phagy during the early phase of Pi deprivation, promoting phospholipid recycling and preventing excessive membrane restructuring [93]. Autophagy-deficient mutants exhibit premature Pi exhaustion and severe growth inhibition, indicating that ER-phagy constitutes a critical adaptive mechanism for Pi-stress tolerance [90].

Collectively, these studies establish autophagy as a pivotal metabolic hub during nutrient starvation, integrating organelle turnover, nutrient remobilization, and energy maintenance. Elucidating these mechanisms provides crucial insights for enhancing nutrient-use efficiency and improving stress resilience in crops cultivated under nutrient-limited environments.

### 3.7. Other Abiotic Stresses

Beyond the stresses described above, autophagy has been implicated in plant responses to oxidative stress and ultraviolet (UV) radiation. For instance, autophagy contributes to maintaining chloroplast integrity under oxidative stress [16]. Collectively, autophagy functions as a central adaptive mechanism against diverse abiotic challenges, including drought, salinity, extreme temperatures, light stress, and heavy metal toxicity. By removing damaged components, recycling metabolites, and preserving cellular homeostasis, autophagy significantly enhances plant stress resilience. Future studies should focus on dissecting the signaling networks governing autophagy under different stress conditions and exploring its interactions with other stress response pathways, such as hormone and ROS signaling, thereby providing a theoretical basis for molecular breeding strategies aimed at improving crop stress tolerance.

Overall, these findings underscore the centrality of autophagy in plant abiotic stress resilience. By coordinating organelle turnover, selective degradation, and metabolic adaptation, autophagy enables crops to maintain growth and productivity under diverse environmental stresses. Understanding these mechanisms provides a theoretical basis for future crop improvement strategies aimed at enhancing multi-stress tolerance.

## 4. Crosstalk: Integrating Autophagy with Hormonal, Metabolic, and Stress Signaling

### 4.1. Interplay Between Autophagy, Cell Death and Immunity

In plant immunity, autophagy functions not merely as a pro-survival module but also as a rheostat that fine-tunes programmed cell death (PCD), thereby balancing defense with growth. This dual role underscores its complexity in dictating the type of cell death, the amplitude of immune output and the progression of senescence. In Arabidopsis, the stress-responsive transcription factor *WRKY33* directly binds the *ATG18a* promoter to activate autophagic flux and strengthen resistance to bacterial pathogens [94]. Conversely, atg mutants accumulate supra-optimal salicylic acid (SA), which fuels unchecked ROS accumulation and runaway cell death, revealing a negative-feedback loop in which autophagy constrains oxidative damage [95]. The plant-specific metacaspase AtMC1 adds another layer of regulation: in young tissues, AtMC1 and autophagy cooperate to promote hypersensitive-response (HR)-type PCD, whereas this cooperation is repressed in older plants, indicating an age-dependent switch in immune strategy [93]. Collectively, these data position autophagy as a guardian of cellular homeostasis that, under biotic stress, calibrates immunity-associated PCD to maximize host fitness.

### 4.2. Convergence of Autophagy with Hormone, ROS and Ca^2+^ Signaling

Autophagy is not an isolated degradative route but an integration hub that intersects with hormonal networks, ROS homeostasis and Ca^2+^ signaling to build a highly connected stress-response circuitry [6,27]. Recent reviews have highlighted that plant hormones modulate autophagy via multiple signaling cascades, coordinating growth, stress response, and nutrient remobilization [96]. This review emphasizes that ABA, auxin, cytokinin, BR, JA, and SA converge on autophagy pathways, linking environmental cues to cellular homeostasis. Abscisic acid (ABA) is a canonical upstream activator: under drought or osmotic stress, ABA induces the AIM-containing protein AtTSPO, which interacts with ATG8 and is subsequently removed by selective autophagy [55]. ABA also suppresses TOR via the SnRK2–RAPTOR branch, thereby licensing autophagosome formation [97]. Additional hormones exert opposing or synergistic control: auxin represses autophagy through the ROP2–TOR axis [98], cytokinin promote the autophagic degradation of type-A ARR proteins to modulate senescence and nutrient remobilization [99], and brassinosteroid (BR) signaling activates autophagic flux under starvation or drought by recruiting the transcription factor BZR1 to ATG promoters [100]. Jasmonic acid (JA) and SA likewise feed into the autophagy circuitry to shape defense and redox poise [94,95,97].

ROS constitute a second master node. Selective autophagy eliminates damaged chloroplasts, mitochondria and peroxisomes, thereby preventing ROS hyper-accumulation and preserving redox balance [101,102]. In atg backgrounds, the ROS–SA feed-forward loop becomes unleashed, triggering PCD, whereas wild-type plants restrain this loop through autophagic clearance [95,103]. Ca^2+^ adds a third dimension: Ca^2+^-dependent protein kinases (CPKs) activate NADPH oxidases to produce an apoplastic ROS burst, which in turn elicits further Ca^2+^ influx, generating a self-propagating Ca^2+^–ROS wave that coordinates local and systemic stress responses [104]. Although direct Ca^2+^-mediated regulation of plant autophagy remains elusive, conserved Ca^2+^/CaM–AMPK modules documented in yeast and mammals provide a plausible mechanistic paradigm [105].

### 4.3. Autophagy in the Regulation of Primary Metabolism and Energy Balance

Autophagy sits at the heart of plant metabolism and energy balance. By dismantling superfluous or damaged organelles, proteins and macromolecules, it releases amino acids, sugars and lipids that can be re-routed into central metabolic pathways [8,10,106]. Under nutrient scarcity or environmental stress, autophagy sustains mitochondrial integrity and ATP production, and facilitates long-distance remobilization of nutrients from source to sink, thereby securing survival and growth [106]. The recycled metabolites feedback on the SnRK1–TOR axis to re-set energy signaling [107]. Nitrogen status, sensed through the HY5–HDA9 module and FLZ proteins, modulates SnRK1–TOR activity, aligning autophagy with C–N metabolic reprogramming and nutrient partitioning [108,109]. Thus, autophagy not only executes degradation but also integrates metabolic and energy cues to optimize plant performance under fluctuating environments.

### 4.4. Autophagy in Organellar Homeostasis: Coupling to Photosynthesis and Respiration

By selectively removing dysfunctional chloroplasts and mitochondria, autophagy preserves the core machinery of photosynthesis and respiration. Under photo-oxidative stress, prolonged darkness or oxidative insults, plants activate chlorophagy and mitophagy to deliver impaired plastids and mitochondria to the vacuole, thereby restricting ROS overflow and safeguarding metabolic homeostasis [28,110,111]. Consistently, *atg5* and *atg7* mutants exhibit reduced photosynthetic efficiency, delayed mitochondrial turnover, hypersensitivity to high-light stress and precocious senescence [112,113]. These phenotypes underscore that autophagy underpins energy organelle quality control, providing the physiological buffer required for growth and stress adaptation.

## 5. Engineering Autophagy for Crop Improvement

### 5.1. Gain-of-Function Strategies: Overexpression of ATG Genes and Receptors

Substantial genetic and physiological evidence demonstrates that enhanced autophagy activity improves plant stress tolerance [31,114]. In *Arabidopsis*, functional ATG pathways are closely associated with an increased tolerance to drought, salinity, and nutrient starvation, whereas *atg5* and *atg7* mutants display premature senescence, growth retardation, and hypersensitivity to carbon or nitrogen deprivation [7,9]. Enhanced autophagic flux helps maintain metabolic balance by clearing damaged cellular components during stress [115].

Functional studies in crops further confirm this trend: overexpression of ATG genes or selective autophagy receptors (e.g., NBR1) has been shown to enhance tolerance to drought and salt stress in several plant species, including apple (MdATG8i, MdATG10) and poplar (NBR1) [37,63,64]. Thus, strengthening autophagy represents a promising strategy for improving crop stress resilience, although gene targets and breeding approaches must be optimized and validated under field conditions [114].

### 5.2. Loss-of-Function and Precision Engineering: Insights from RNAi and CRISPR/Cas9

RNAi-mediated silencing of autophagy-related genes has provided direct functional evidence for the essential role of autophagy in stress adaptation. In *Arabidopsis*, loss-of-function mutants of *ATG5* or *ATG7* exhibit premature senescence, defective nutrient remobilization, and hypersensitivity to carbon, nitrogen, and drought stress [14,116]. Similarly, disruption of *OsATG7* in rice leads to impaired autophagic activity, reduced biomass, and enhanced sensitivity to drought and nutrient starvation, confirming the conserved role of autophagy in maintaining stress resilience [30].

CRISPR/Cas9 genome editing has become an effective tool to study autophagy genes in plants. Knockout or multiplex editing of ATG family members enables functional dissection of autophagy in stress responses. For example, *SlATG5* mutants generated by CRISPR showed reduced drought tolerance and accelerated senescence in tomato [117], and *ATG6* editing in petunia impaired nutrient remobilization and seed yield [118]. These findings confirm that autophagy contributes to stress adaptation and productivity. Most current applications remain at the functional-analysis stage, and further integration with field evaluation is required for crop improvement [119].

### 5.3. Synthesis and Future Directions: Pathways to Field Application

Targeted regulation of the autophagy pathway has significant potential to enhance crop stress tolerance, nutrient use efficiency, and senescence control, making autophagy an attractive target for molecular breeding and biotechnological innovation [30,119]. Across diverse crops and model species, induction of ATG expression and increased autophagic flux under stress have been consistently associated with enhanced tolerance to drought, salinity, and heat [36,81,120,121]. Conversely, atg mutants exhibit hypersensitivity to abiotic stress, underscoring the essential role of autophagy in stress adaptation [6,117]. Overexpression of *ATG* genes or selective autophagy receptors such as NBR1 has been shown to improve drought resistance, antioxidant capacity, and water-use efficiency in crops including apple, tomato, and wheat, highlighting the functional transferability of autophagy-based strategies [121].From a breeding perspective, autophagy manipulation can be approached from four complementary directions: (i) flux enhancement, by overexpressing or activating ATG genes to boost autophagic activity [36,121]; (ii) selective autophagy regulation, by exploiting receptor proteins to fine-tune intracellular cargo clearance [122]; (iii) genome editing and flux tuning, by applying CRISPR-based tools to optimize ATG promoters or regulatory elements [123]; and (iv) integration with nutrient-use and quality improvement, combining autophagy engineering with conventional breeding to simultaneously improve yield and crop quality [119].

### 5.4. Future Perspectives and Synthetic Biology Approaches

While the strategies summarized in 5.3 demonstrate the potential of autophagy engineering, future approaches could leverage advanced synthetic biology tools to achieve more precise, controllable, and context-specific regulation. CRISPRa/i systems enable stress- or tissue-specific activation or repression of autophagy genes, while synthetic promoters can drive modular and inducible expression of ATG circuits. Modular ATG gene circuits could coordinate multiple autophagy components in response to environmental cues. In addition, inducible or optogenetic systems may allow temporal fine-tuning of autophagic activity to minimize unintended effects.

Several challenges must be addressed to translate these strategies to crop improvement. Tissue- or cell type-specific control is essential to avoid compromising growth, while minimizing off-target effects is critical for both functional fidelity and biosafety. Balancing growth–defense trade-offs remain a major concern, as excessive autophagy activation may reduce biomass accumulation. Furthermore, complex and fluctuating field conditions cannot be fully recapitulated in controlled laboratory experiments, highlighting the need for rigorous field validation.

Potential crop traits most likely to benefit from engineered autophagy include drought tolerance, salt and heat stress resilience, improved nutrient remobilization, delayed senescence, and enhanced yield or quality. Integrating autophagy engineering with conventional breeding, high-throughput phenotyping, and precision agriculture technologies will likely be necessary to achieve robust and sustainable improvements under field conditions. Addressing these aspects provides a clear roadmap for translating laboratory discoveries into practical crop improvement strategies and sets the stage for the rational design of next-generation autophagy-based interventions.

## 6. Multi-Omics Approaches to Dissect Autophagy Mechanisms

### 6.1. Transcriptomics and Prospective Epigenomic Insights

High-throughput transcriptomic analyses, notably bulk RNA-seq, have been instrumental in revealing how ATG genes and selective autophagy receptors respond to abiotic stresses, and how autophagy broadly modulates stress-responsive gene networks. For example, comparative transcriptome profiling of Arabidopsis wild-type and atg5 mutant plants under heat stress showed massive differential expression of genes involved in metabolism, hormone signaling, stress responses, DNA repair and other cellular processes, indicating that autophagy influences heat stress responses beyond protein degradation alone [124]. In addition, natural-accession studies under nitrate starvation demonstrated that most ATG genes are transcriptionally upregulated when nitrogen is limiting, highlighting transcriptional plasticity of the autophagy pathway under nutrient stress [125]. Similar expression induction of multiple ATG family members under low-nitrogen stress has also been reported in crop species, e.g., sugar beet [126], underscoring the relevance of transcriptomic approaches in both model plants and crops. Beyond stress conditions, integrative transcriptome–metabolome analyses in Arabidopsis revealed that autophagy significantly affects primary metabolism and development, supporting the notion that multi-omics provides a holistic view of autophagy’s roles in cellular homeostasis [127]. Although direct epigenomic data (e.g., DNA methylation or histone modification at ATG loci) remain scarce in plants, combining transcriptomics with future epigenomic profiling could reveal upstream regulators and chromatin-level mechanisms of ATG gene regulation under stress.

### 6.2. Proteomics and Post-Translational Modifications

Proteomic approaches enable quantification of ATG proteins, selective autophagy receptors (e.g., NBR1), and their interacting partners in planta, thereby revealing how autophagy shapes the proteome under stress or in mutant contexts. In particular, in vivo protein turnover studies using nitrogen-15 (^15^N) labeling in Arabidopsis atg5 and atg11 mutants have demonstrated that more than 100 proteins show markedly slower degradation in autophagy-deficient backgrounds, indicating both canonical and noncanonical autophagy cargo [128]. These data provide direct mechanistic insights into how selective autophagy contributes to protein homeostasis at the proteome level.

Moreover, post-translational modifications (PTMs) such as phosphorylation, ubiquitination, and acetylation are increasingly recognized as pivotal regulators of autophagy proteins. For instance, BAK1-mediated phosphorylation of ATG18a suppresses autophagy in Arabidopsis, suggesting that phosphorylation can negatively regulate autophagy under certain conditions [129]. In addition, the acetyltransferase HOOKLESS1 (HLS1) directly acetylates ATG18a under nutrient starvation, and this acetylation enhances the ATG2–ATG18a interaction and the binding of ATG18a to phosphatidylinositol-3-phosphate (PtdIns(3)P), thereby promoting autophagosome formation [130]. Beyond these, a broader survey of PTMs on plant ATG proteins highlights that ubiquitination (e.g., via SINAT E3 ligases) and phosphorylation (e.g., by SnRK1 or TOR-related kinases) modulate key ATG components, affecting their stability and activity [131].

### 6.3. Metabolomics and Lipidomics

Metabolomic and lipidomic profiling complement proteomic analyses by revealing the biochemical consequences of altered autophagy activity. In Arabidopsis, autophagy-deficient mutants display significant changes in amino acids, sugars, secondary metabolites, and redox-related metabolites, reflecting impaired metabolic recycling and energy homeostasis [132]. Lipidomic studies further demonstrate substantial remodeling of phospholipids, sphingolipids, and galactolipids in atg mutants, highlighting the critical role of autophagy in membrane maintenance and organelle integrity under stress. These lipid alterations are consistent with functional disruptions of chloroplasts and other organelles, underscoring the central role of autophagy in cellular homeostasis [132].

Moreover, complementary metabolomic analyses in Arabidopsis and crop species have shown that autophagy impacts not only lipid metabolism but also nitrogen and carbon-related metabolites, such as amino acids, organic acids, and sugars, further linking autophagic activity to energy balance and stress adaptation [127]. Collectively, these studies indicate that integrative metabolomic and lipidomic profiling provides a holistic view of how autophagy sustains metabolic and organelle homeostasis under adverse conditions.

### 6.4. Systems Biology Integration

The integration of transcriptomic, proteomic, metabolomic, and lipidomic datasets provides a systems-level perspective on the regulatory architecture underlying stress-induced autophagy. By capturing coordinated changes in gene expression, protein abundance and turnover, metabolic fluxes, and lipid remodeling, multi-omics analyses reveal regulatory relationships that are not evident from single-layer datasets. Recent advances in multi-omics network inference have established robust methodological frameworks for constructing condition-specific regulatory maps, enabling the identification of upstream regulators, signaling nodes, and feedback circuits that coordinate autophagy with broader abiotic stress-response pathways [133,134]. Incorporating spatial and temporal resolution—through single-cell profiling, tissue-specific omics, and time-course analyses—further refines our ability to delineate autophagic flux dynamics and distinguish early regulatory events from downstream metabolic consequences.

Computational modeling pipelines, including modular clustering algorithms and probabilistic graphical approaches, facilitate systematic prediction of regulatory interactions and potential master regulators linking environmental inputs to ATG gene activation. Integrated transcriptomic–metabolomic studies of abiotic stress responses have demonstrated the power of these approaches in reconstructing regulatory modules and identifying stress-dependent shifts in cellular metabolic states [134]. Together, these systems-level analyses provide a conceptual and technical foundation for defining autophagy-centered regulatory networks and for guiding targeted mechanistic validation in diverse plant species.

## 7. Future Perspectives

Over the past decade, remarkable progress has been made in understanding plant autophagy; however, many fundamental questions remain unresolved [8]. For example, the precise mechanisms by which autophagy enhances plant stress tolerance are not fully understood. Future studies should aim to identify the key autophagic substrates degraded during stress responses and systematically dissect the regulatory networks connecting autophagy with other stress signaling pathways.

Despite these advances, the full range of autophagy’s roles under diverse environmental conditions remains to be fully elucidated. Future efforts should focus on uncovering plant-specific regulatory pathways and molecular networks governing autophagy. Such discoveries will provide novel strategies for enhancing stress resilience and productivity in crops [135,136].

### 7.1. Research Gaps and Priority Areas

Despite substantial progress in defining the core machinery and physiological relevance of autophagy, several critical gaps limit our understanding of how autophagy integrates into plant stress responses [8,27]. Addressing these gaps is essential for constructing predictive frameworks and enabling targeted manipulation of autophagy for crop improvement.

**Autophagy under combinatorial stresses:** Most studies have focused on single stress factors such as drought, salinity, or nutrient deprivation. In natural and agricultural environments, however, plants frequently face simultaneous or sequential stresses. The mechanisms by which distinct stress signaling pathways converge on the autophagy network remain largely unclear. It is also unknown whether autophagy functions synergistically, antagonistically, or hierarchically under combined stress conditions [55,97]. Understanding these interactions is crucial for translating autophagy research into field-relevant applications.

**Plant-specific autophagy regulators and upstream sensors:** Key regulators, including TOR, SnRK1, and heat-stress factors, have been partially characterized. Nevertheless, many plant-specific modulators remain unidentified. These include membrane-shaping proteins, ATG8-interacting receptors, nutrient sensors, and redox-responsive factors [8,95]. Discovering these elements is essential to establish stimulus-specific regulation of autophagy and to understand how plants integrate environmental cues with intracellular degradation pathways.

**Organ- and cell type–specific autophagy dynamics:** Autophagy exhibits strong spatial specificity, with distinct dynamics in roots, reproductive tissues, vasculature, guard cells, and meristems. Comprehensive studies examining tissue- and cell type-specific regulation are still lacking. Without this knowledge, it is difficult to understand how local autophagic responses contribute to whole-plant adaptation, resource allocation, and developmental progression [101,102].

**Crosstalk between autophagy and hormone signaling under abiotic stress:** Numerous phytohormones—including ABA, brassinosteroids, cytokinins, auxin, jasmonates, and salicylic acid—modulate autophagy. Yet the hierarchical relationships, spatiotemporal interactions, and stress-specific regulatory pathways remain largely unresolved [55,97,98]. Clarifying these interactions is a major step toward integrating autophagy into broader stress signaling frameworks.

**Selective autophagy substrates and recognition mechanisms:** Although selective processes such as chlorophagy, mitophagy, ER-phagy, and pexophagy have been described, the full catalog of stress-induced autophagy cargos is incomplete. The molecular determinants that guide selective recognition are also poorly defined [95,101,102,103]. Mapping these substrates and their recognition mechanisms is essential to understand how autophagy prioritizes cellular components under stress and nutrient-limiting conditions.

**Integrative and predictive models of autophagy:** Autophagy responds dynamically to metabolic cues, redox status, and environmental fluctuations. However, most studies remain descriptive. Developing time-resolved multi-omics datasets, quantitative imaging approaches, and computational modeling will be crucial to construct predictive models of autophagy behavior under complex and fluctuating environmental conditions.

### 7.2. Targeted Gene Regulation

Future research should focus on the targeted manipulation of autophagy-related genes to enhance crop stress tolerance. By identifying key regulatory factors of autophagy and developing crops with elevated autophagic activity, it will be possible to generate resilient varieties capable of thriving under harsh environmental conditions. In the context of climate change, this approach offers considerable promise for improving crop yield and safeguarding global food security.

### 7.3. Integration of Multi-Omics Approaches

The integration of genomics, transcriptomics, proteomics, and metabolomics will provide a comprehensive understanding of the molecular mechanisms by which autophagy mediates plant stress responses. By elucidating the complex regulatory networks controlling autophagy, researchers can identify novel targets for crop improvement and develop innovative strategies to enhance stress resilience.

### 7.4. Engineering Synthetic Autophagy Pathways

The development of synthetic autophagy pathways presents a promising avenue for enhancing crop stress tolerance. By constructing engineered pathways, autophagic activity can be precisely and controllably modulated, providing a powerful tool for crop improvement. This approach has the potential to cultivate crops that are resilient to climate change and capable of withstanding multiple environmental stresses, thereby transforming agricultural practice.

### 7.5. Field Trials and Validation

Conducting field trials to evaluate the performance of autophagy-enhanced crops under real-world conditions is critical for translating research findings into practical applications. Field testing allows for the assessment of crop performance across diverse environmental scenarios and the selection of varieties with the highest commercial potential. This step is essential to ensure the successful implementation of autophagy-based agricultural strategies.

### 7.6. Crosstalk Between Autophagy and Other Stress Response Pathways

Understanding the crosstalk between autophagy and multiple signaling networks remains a key frontier. Current evidence indicates that autophagy is regulated by hormones such as ABA, BR, and auxin, and interacts with ROS homeostasis and Ca^2+^ signaling to modulate stress tolerance [95,97]. However, the hierarchical relationships and dynamic regulation of these signals under multifactorial stress conditions remain unclear. Future studies should employ spatiotemporally precise genetic tools and multi-omics approaches to dissect the core nodes of autophagy–signal interactions, and to explore strategies for coordinating autophagy with hormonal, ROS, and Ca^2+^ signaling to drive crop stress resilience and molecular breeding.

### 7.7. Adoption of Climate-Smart Agriculture

Implementing climate-smart agricultural practices that leverage the potential of autophagy to enhance crop stress tolerance is crucial for ensuring food security under changing climatic conditions. Integrating autophagy-based strategies into agricultural systems will enable the development of resilient and sustainable food production frameworks capable of meeting climate-related challenges. This approach is essential for securing long-term agricultural sustainability and global food security.

## 8. Conclusions

Autophagy is a central adaptive mechanism in plants, essential for maintaining cellular homeostasis, coordinating stress responses, and optimizing resource allocation under diverse abiotic stresses, including drought, salinity, extreme temperatures, and oxidative challenges. Core ATG proteins, selective autophagy receptors, and regulatory complexes such as TOR and SnRK1 orchestrate the precise control of autophagosome formation, cargo recognition, and degradation, enabling plants to adjust rapidly and flexibly to environmental fluctuations.

This review highlights the pivotal role of autophagy in mitigating cellular damage, supporting nutrient remobilization, regulating development, and fine-tuning stress-responsive gene expression. The integration of autophagy with hormonal, ROS, and nutrient signaling pathways underscores its function as a hub linking cellular maintenance to adaptive strategies.

From an applied perspective, engineering autophagy through genetic, chemical, or synthetic biology approaches offers a promising avenue for improving crop stress tolerance and productivity. This manuscript synthesizes current knowledge on autophagy mechanisms under abiotic stress, identifies key research gaps, and outlines potential strategies for crop improvement, providing a comprehensive framework for future studies and translational applications.

## Data Availability

No new data were created or analyzed in this study. Data sharing is not applicable to this article.

## Abbreviations

The following abbreviations are used in this manuscript:

ABA	Abscisic acid
AMP	Adenosine monophosphate
AMPK	AMP-activated protein kinase
AOX1a	Alternative oxidase 1a
As	Arsenic
ATG	Autophagy-related
BR	Brassinosteroid
BRI1a	Brassinosteroid insensitive 1a
BZR1	Brassinosteroid insensitive 1
Cd	Cadmium
CPKs	Ca^2+^-dependent protein kinases
CRISPR/Cas9	Clustered regularly interspaced short palindromic repeats/CRISPR-associated protein 9
EIN3/EIL1	Ethylene-insensitive 3/ethylene-insensitive-like 1
ER	Endoplasmic-reticulum
ER-phagy	Endoplasmic-reticulum autophagy
GABARAP	GABA(A) receptor-associated protein
HIPP33	Heavy metal-associated isoprenylated plant protein 33
HR	Hypersensitive-response
HY5–HDA9	HY5 (Long Hypocotyl 5)—Histone Deacetylase 9
JA	Jasmonic acid
LAMP-2	Lysosomal-associated membrane protein 2
LC3	Microtubule-associated protein 1A/1B-light chain 3
mTOR	Mammalian target of rapamycin
MtPIP2;7	Plasma membrane intrinsic protein 2;7
NBR1	Neighbor of BRCA1 gene 1
NCOA4	Nuclear receptor coactivator 4
Ni	Nickel
O-MDH3-ATI1	O-Malate Dehydrogenase 3—Associated Transcription Initiator 1
PAS	Phagophore-assembly site
PCD	Programmed cell death
PE	Phosphatidylethanolamine
PI3K	Class-III phosphatidylinositol 3-kinase
PINK1	PTEN-induced kinase 1
PI3P	Phosphatidylinositol 3-phosphate
Rab7	Rab GTPase subfamily member 7
ROS	Reactive oxygen species
SA	Salicylic acid
SnRK1	Sucrose-non-fermenting-1-related kinase 1
SnRK2	Sucrose-non-fermenting-1-related kinase 2
SNARE	Soluble N-ethylmaleimide-sensitive factor Attachment protein REceptor
STX17	Syntaxin 17
TGA	Transcriptional activation factor
TOR	Target-of-rapamycin
Ufm1	Ubiquitin-fold modifier 1
Ufl1	Ufm1-activating enzyme 1
UPR	Unfolded-protin response
UV	Ultraviolet
WRKY53	WRKY53 WRKY transcription factor 53
WRKY33	WRKY33 WRKY transcription factor 33
